# Food Identity, Authenticity and Fraud: The Full Spectrum

**DOI:** 10.3390/foods6070049

**Published:** 2017-07-06

**Authors:** Saskia M. van Ruth, Daniel Granato

**Affiliations:** 1 Food Quality and Design Group/RIKILT, Wageningen University and Research, P.O. Box 230, 6700 AE Wageningen, The Netherlands; 2 State University of Ponta Grossa, Av. Carlos Cavalcanti, 4748, Uvaranas Campus, 84030-900 Ponta Grossa, PR, Brazil; dgranato@uepg.br

We are pleased to introduce this Special Issue of *Foods* dedicated to ‘Food Identity, Authenticity and Fraud: The Full Spectrum’. This Special Issue concentrates on various food authenticity and fraud topics and allows researchers to share, present and discuss their latest developments and innovations with a global audience. Food fraud is a global issue which damages the reputation of companies, disrupts markets and erodes consumer confidence. Food fraud surfaces more frequently in certain supply chains. The six most frequently reported product groups in three international fraud databases are spices and herbs, olive oil, seafood, dairy products, meat, and other oils and fats [1]. The importance of studies covering these topics is mainly related to economic issues associated with frauds of high-value added foods with cheaper ingredients. For instance, adulterating a cheese with Protected Designation of Origin with a conventional cheese or marketing ground coffee as organic without certification, among other examples. To mitigate food fraud, knowledge on why and how food fraud is happening is vital. Furthermore, technical and managerial control measures are needed in fraud vulnerable situations [2]. The technical control measures include analytical tests which need to be improved and updated all the time since fraudsters will always try to catch up and circumvent these methods. In the past, analytical methods were primarily focused on the detection of single compounds, e.g., protein or fat content in milk. Later on, multiple compounds or ratios of compounds were considered. In the past decade, we have seen an increase in methods which consider a full spectrum or full chromatogram as an analytical fingerprint of a food. Due to the complexity of the data, comparison of these fingerprints requires more advanced statistical modelling techniques. Although there used to be the tendency to aim for the detection of individual adulterants, a trend of assuring the general authenticity of a product is emerging. In this case, authentic material is separated from any unusual material to allow rapid confirmation of the authenticity, which is also called broad anomaly testing. This more general screening for the good and the bad is also seen with the miniaturization of instruments. Portable, handheld devices are very suitable for a first impression of a food product and for red/green traffic light approaches. Although fraud detection will not prevent fraud, fraud detection is an inseparable part of fraud mitigation.

In the current Special Issue, novel fraud detection methods are presented. They deal with a number of fraud vulnerable commodities, such as seafood [3], olive oil [4], and dairy products [5,6]. Fraud categories covered include compositional fraud, for instance replacement of olive oil with other vegetable oils or the addition of egg yolk to sea urchin roe. Furthermore, they include fraud within production systems and with regards to the geographical origin/product typicality. The scientists that contributed to this Special Issue report on novel methods based on color, non-volatile compounds, and fat constituents. Three of the studies deal with fingerprint methods that combine analytical techniques and chemometrics, and one study deals with a simple but effective color measurement for use beyond the laboratory. We acknowledge the efforts of the authors of the publications of this Special Issue. Their contributions will help to shape the future of food authentication.

## References

[1] Weesepoel Y.J.A., van Ruth S.M. (2015). Inventarisatie van Voedselfraude: Mondiaal Kwetsbare Productgroepen en Ontwikkeling van Analytische Methoden in Europees Onderzoek.

[2] Van Ruth S.M., Huisman W., Luning P.A. (2017). Food fraud vulnerability and its key factors. Trends Food Sci. Tech..

[3] Meloni D., Spina A., Satta G., Chessa V. (2016). A Rapid Colorimetric Method Reveals Fraudulent Substitutions in Sea Urchin Roe Marketed in Sardinia (Italy). Foods.

[4] Messai H., Farman M., Sarraj-Laabidi A., Hammami-Semmar A., Semmar N. (2016). Chemometrics Methods for Specificity, Authenticity and Traceability Analysis of Olive Oils: Principles, Classifications and Applications. Foods.

[5] Pustjens A.M., Boerrigter-Eenling R., Koot A.H., Rozijn M., van Ruth S.M. (2017). Characterization of Retail Conventional, Organic, and Grass Full-Fat Butters by Their Fat Contents, Free Fatty Acid Contents, and Triglyceride and Fatty Acid Profiling. Foods.

[6] Popping B., De Dominicis E., Dante M., Nocetti M. (2017). Identification of the Geographic Origin of Parmigiano Reggiano (P.D.O.) Cheeses Deploying Non-Targeted Mass Spectrometry and Chemometrics. Foods.

